# Cardiopulmonary coupling-calculated sleep stability and nocturnal heart rate kinetics as a potential indicator for cardiovascular health: a relationship with blood pressure dipping

**DOI:** 10.3389/frsle.2024.1230958

**Published:** 2024-08-01

**Authors:** Hugi Hilmisson, Robert Joseph Thomas, Solveig Magnusdottir

**Affiliations:** ^1^MyCardio LLC, SleepImage^®^, Denver, CO, United States; ^2^Department of Medicine, Division of Pulmonary, Critical Care and Sleep Medicine, Beth Israel Deaconess Medical Center, Boston, MA, United States

**Keywords:** sleep stability, blood pressure dipping, cardiovascular disease, cardiopulmonary coupling (CPC), heart rate kinetics, heart rate dipping

## Abstract

**Introduction:**

High blood pressure (HBP) is an independent, modifiable driver of cardiovascular (CV) morbidity and mortality. Nocturnal hypertension and non-dipping of blood pressure (NdBP) may be early markers of HBP. Similar to patients with NdBP, individuals with non-dipping of heart rate (NdHR) during sleep have an increased risk of CV disease, CV events, and CV-related mortality. The aim of this analysis was to evaluate if cardiopulmonary coupling (CPC) analysis-derived sleep states [stable/unstable non-rapid eye movement (NREM) sleep] and concomitant heart rate (HR) changes can provide information about nocturnal blood pressure (BP).

**Method:**

Plethysmogram (pleth) signals from the HeartBEAT study (NCT01086800) were analyzed for CPC sleep states. Included in the analysis are sleep recordings from participants with acceptable pleth-signal quality at baseline (*n* = 302) and follow-up (*n* = 267), all having confirmed CV disease or CV-disease risk factors. The participants had a high prevalence of obstructive sleep apnea (OSA), 98.4% with moderate-OSA [apnea–hypopnea index (AHI) ≥ 15) and 29.6% severe OSA (AHI ≥ 30). A “heart-rate module” was created to evaluate the utility of identifying patients more likely to have BP dipping during sleep. Patients who did not have a decrease of ≥10% in their BP from wake to sleep were defined as NdBP and NdHR if their heart rate during stable-NREM sleep was higher than during unstable-NREM sleep.

**Results:**

The most significant difference in minimum HR (HR_min_) was observed when comparing BP dippers [56 ± 4 beats per minute (BPM)] and non-BP dippers (59 ± 4 BPM; *p* < 0.0001) during diastolic blood pressure in stable-NREM sleep. Higher HR_min_ were associated with an increased likelihood of being a non-dipper, with the strongest relationship with diastolic BP and stable-NREM sleep. Every increase of 1 BPM during stable-NREM sleep was associated with an ~4.4% increase in the probability of NdBP (*p* = 0.001). Subjects with NdHR have higher mean BP during sleep and wake periods than HR dippers. When continuous positive airway pressure therapy is efficacious, and a dipping pattern is achieved—physical and mental health is improved.

**Conclusion:**

HR analytics in relation to the sleep period and the CPC spectrogram-estimated sleep states can provide novel and potentially clinically useful information on autonomic health. HR dipping (or not) may be a useful screener of BP dipping or non-dipping to identify individuals who may benefit from a formal assessment of 24-h ambulatory BP. Such a stepped approach may enable a more practical and applicable approach to diagnosing HBP.

**Clinical Trial Registration:**

The Heart Biomarker Evaluation in Apnea Treatment (HeartBEAT) study is registered at clinicaltrials.gov/ct2/show/NCT01086800.

## 1 Introduction

High blood pressure (HBP) is a major independent and modifiable driver of cardiovascular (CV) morbidity and mortality globally (Mills et al., 2020). Therefore, early diagnosis and optimal management of HBP are essential (Williams et al., 2018). Hypertension may be overt, with the physician's estimate and multiple home recordings in agreement, or it may be less obvious. The term *masked hypertension* (MH) was first introduced in 2002, describing a hypertension phenotype characterized by normal/not-increased office blood pressure (BP) readings and elevated out-of-office BP (Pickering et al., 2002). Identifying the MH phenotype is of interest for multiple reasons: (a) the prevalence is substantial, estimated at ~10%−30% of individuals attending hypertension clinics (O'Brien et al., 2013; Stergiou et al., 2021); (b) MH increases the risk of CV morbidity, with a meta-analysis comparing normotensive individuals and those with MH finding a 2.09 times increase in experiencing CV events for individuals with MH, while individuals with sustained hypertension have a 2.59 times increase in CV events (Pierdomenico and Cuccurullo, 2011); and (c) MH is more difficult to diagnose, as an out-of-office evaluation is required with 24-h ambulatory BP monitoring (24-ABPM). The availability of classic cuff 24-ABPM may be relatively limited and expensive, and the process is cumbersome and uncomfortable for the user, with cuff inflations and the frequency of measurements possibly causing arousals, disturbing sleep, and affecting the nighttime BP (NBP) measurement, which may not represent the true NBP (Pickering et al., 2006). While novel technologies are in development, including wrist pulse wave analyses, these are not yet fully validated. Moreover, such new technologies enable the collection of a large number of BP readings, which are not readily transferable to intermittent cuff inflation. Thus, disease-related validation remains to be done with more continuous forms of BP recording.

Nocturnal hypertension and non-dipping of BP may be early markers of HBP and MH. Nocturnal BP and night-to-day BP ratio have been identified as significant predictors of adverse CV outcomes (O'Brien et al., 1988; Asayama et al., 2023) and better predictors of fatal and non-fatal CV events and organ damage than daytime BP (Ohkubo et al., 2002; Salles et al., 2016; Staplin et al., 2023). Similar to changes in NBP, individuals with non-dipping of their heart rate (HR) during sleep have an increased risk of CV disease, CV events, and CV-related mortality (Eguchi et al., 2009; Kabutoya et al., 2010; Tadic et al., 2018; Nelde et al., 2023). For example, obstructive sleep apnea (OSA) is a common disease capable of disrupting normal BP control (Senaratna et al., 2017; Benjafield et al., 2019). A bidirectional relationship exists between OSA and HBP, with OSA patients having an increased risk of developing HBP, and the prevalence of OSA is higher in patients with HBP (Sawatari et al., 2016). However, other causes of sleep disruption such as insomnia and periodic limb movements are also associated with elevated NBP (Palagini et al., 2013).

Current guidelines highlight the importance of accurate diagnosis of HBP. The gold standard for identifying MH is 24-ABPM (Franklin et al., 2017), but still, the diagnosis is largely based on in-office BP measures, and patients with HBP and MH may therefore remain undiagnosed (Stergiou et al., 2021). Somewhat surprisingly, another readily available signal, HR during sleep [from ambulatory recordings and the millions of laboratory polysomnography (PSG) or home polygraphy done yearly], has not been subjected to rigorous analysis. Arousals from sleep are associated with both HR and BP elevations. It is thus plausible that an analysis of HR during sleep may provide a surrogate of BP or at least identify those who should have a formal 24-ABPM performed. HBP in patients with OSA is often predominantly nocturnal, and non-dipping BP is common (Marin et al., 2012). Therefore, alternative, simple, and less invasive measures that may estimate changes related to HR might be of value to identify non-dippers for further evaluation, including 24-ABPM as a tool that might assist in managing patients with OSA, insomnia, or comorbid insomnia and sleep apnea (Sweetman et al., 2019) and HBP (Tadic et al., 2018; Nelde et al., 2023).

This report targeted nocturnal heart rate (NHR) by analyzing photoplethysmogram (PPG) and oximetry information (SpO_2_) recorded during a conventional home sleep apnea test (HSAT). The cardiopulmonary coupling (CPC) sleep state and the HR kinetics analysis were computed to estimate alignments of HR with stable- and unstable-NREM sleep (Al Ashry et al., 2021b). It has been noticed that HR profiles across the night can on average drop (“HR dipping”), remain relatively flat (“HR non-dipping”), or even rise (“HR reverse dipping”). HR dipping is maximal during stable-NREM sleep periods [high-frequency coupling (HFC)] but can sometimes rise during such periods, suggesting increased sympathetic drive when normally NREM3/stable NREM2 should be associated with reduced sympathetic drive. We hypothesized that a simple method for calculating HR changes during stable-NREM sleep can be utilized to estimate dipping, non-dipping, or reverse-dipping HR. To test our hypothesis, we utilized the Heart Biomarker Evaluation in Apnea Treatment (HeartBEAT) study, which was designed to evaluate alternative approaches to reduce the risk of heart diseases. This study included classic cuff ABPM (Gottlieb et al., 2014).

## 2 Materials and methods

### 2.1 Study design

The HeartBEAT study (NCT01086800) was a four-site, randomized, parallel-group trial among patients with high CV risk (Gottlieb et al., 2014). At baseline, patients were screened for OSA with the Berlin questionnaire (Netzer et al., 1999), and if they were at risk for OSA, an HSAT was initiated utilizing a portable sleep monitor, including recording PPG-signal and pulse-oximetry (SpO_2_) data from a fingertip sensor. Patients with an AHI of ≥15 events per hour of sleep were eligible to participate in the study. Patients with an AHI of >50 and a central index of >5 were excluded from randomization. The primary outcome of the HeartBEAT study was to evaluate changes in 24-h mean arterial blood pressure (MAP). In addition, nocturnal dipping and non-dipping of BP were reported, with non-dipping BP defined as a mean nocturnal BP higher than 90% of the mean daytime BP value. Participants in the HeartBEAT study were randomly assigned to one of three groups: continuous positive airway pressure (CPAP) therapy, nocturnal oxygen therapy, or healthy lifestyle with sleep education. Institutional review board approval was obtained from each participating institution. For this analysis, a data user agreement was obtained from the National Sleep Research Resource (Dean et al., 2016).

### 2.2 Participants

Patients aged 45–75 with established coronary heart disease or multiple CV-disease risk factors and well-managed hypertension, were recruited from cardiology practices at four participating medical centers. Patients with an AHI in the range of 15–50 were offered the opportunity to participate in the study. This study is based on a CPC analysis of the data derived from the fingertip PPG-sensor (the pleth waveform and oxygen data), as well as information reported about participants' dipping or non-dipping status.

### 2.3 Follow-up

A detailed description of the methodology and primary results of the trial's outcome have been reported (Gottlieb et al., 2014). In brief, outcomes were measured at baseline and 12 weeks after randomization. The primary outcome was 24-h MAP (measured using the 90207 Ambulatory Blood Pressure Monitor, Spacelabs Healthcare). The mean pressure was calculated at each reading as one-third of systolic pressure plus two-thirds of diastolic pressure. The 24-h mean pressure was calculated as a weighted average of the mean pressure during wakefulness and sleep, with the weights determined by the percentage of reported time spent in each state as recorded in a sleep diary. Nocturnal non-dipping blood pressure was defined as the mean nocturnal BP higher than 90% of the mean daytime value.

### 2.4 Methods

#### 2.4.1 The data set

The HeartBEAT study measured and reported BP dipping and non-dipping. Subjects were stratified into BP dippers and BP non-dippers, where non-dippers did not demonstrate a decrease in BP of ≥10% from wake to sleep.

#### 2.4.2 CPC analysis

**Figure 1:**
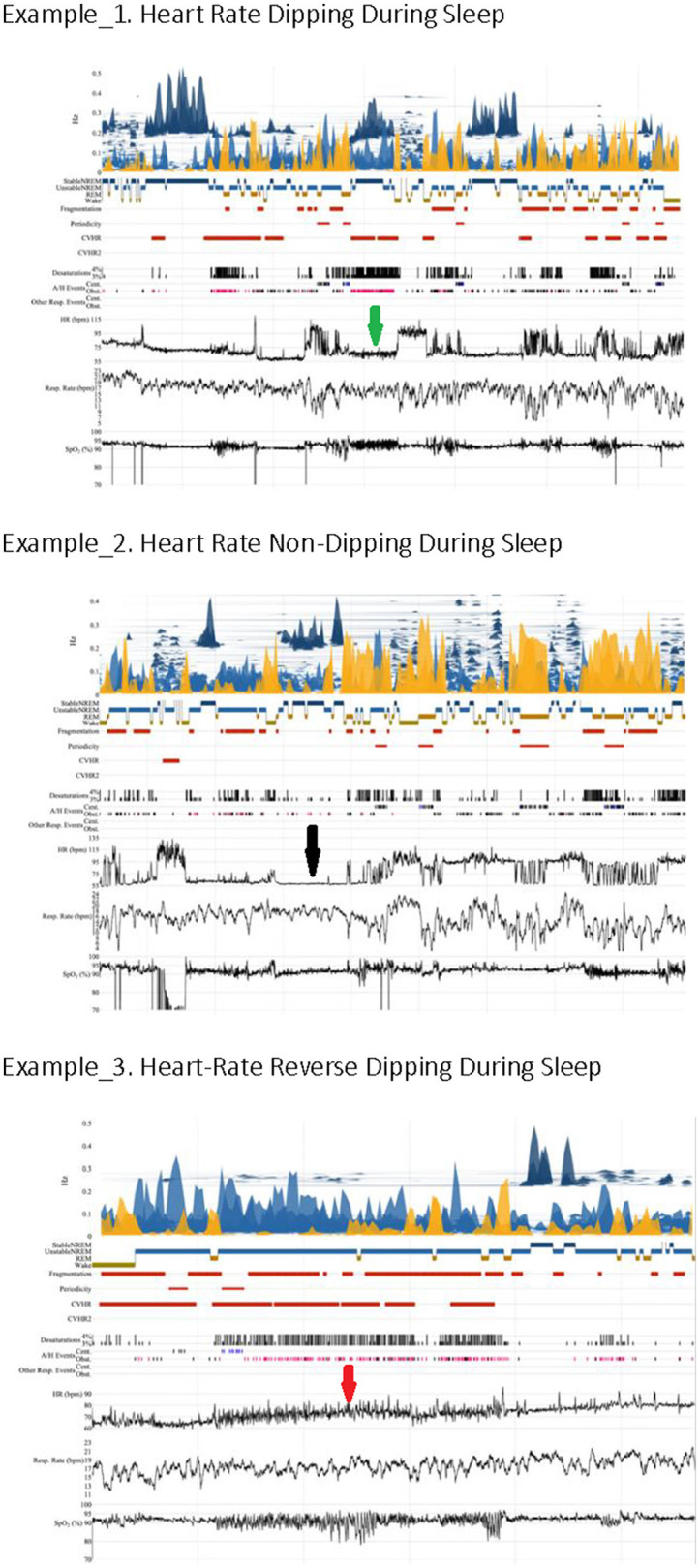
Heart rate kinetics during sleep. The arrows indicate heart rate characteristics (green: dipping, black: non-dipping, red: reverse dipping).

The CPC method has been described in detail in several prior publications (Thomas et al., 2005; Al Ashry et al., 2021a). Cardiopulmonary sleep spectrograms were first obtained from a single lead electrocardiogram (ECG). ECG-derived respiration (EDR) is obtained either by using R-S wave amplitudes or variations in the QRS complex area. Ectopic beats are identified and removed, normal sinus–normal sinus (NN) intervals are extracted, and outliers are filtered (Thomas et al., 2005). After extracting the NN interval series on ECG and its associated EDR, the signals are then resampled using cubic splines at 2 Hz. The fast Fourier transform is applied to three overlapping 512-sample sub-windows within the 1,024-coherence window. The 1,024-coherence window is then advanced by 256 samples (2.1 min), and the calculation is repeated until the entire NN interval/EDR series is analyzed. Thus, the cross-spectral power and coherence of these two signals are calculated over a 1,024-sample (8.5-min) window. For each 1,024-sample window, the product of the coherence and cross-spectral power is used to calculate the ratio of coherent cross-power in the low-frequency (0.01–0.1 Hz) band to that in the high-frequency (0.1–0.4 Hz) band. The logarithm of the high-to-low-frequency CPC ratio [log (HFC/LFC)] is then computed to yield a continuously varying measure of CPC sleep stability/instability output metrics. While, originally, the ECG signal was used as input, any signal or signal set that encodes respiration and heart rate variability (HRV) may be used to compute the CPC sleep spectrogram; most conveniently, this signal set can be obtained from the peripheral PPG-signal, which is readily available from current generation oximeters. The current embodiment uses a ring- or fingertip-based oximeter to collect the data coupled with a mobile application and Bluetooth to stream the data for automated analysis. The SleepImage system complies with the Health Insurance Portability and Accountability Act (HIPAA), is cleared by the Food and Drug Administration (K182618), and complies with the EU Medical Device Directive (CE-mark 2862) to automatically generate biomarkers, presented numerically and graphically (Figure 1). The analysis is otherwise essentially identical. The outputs of the CPC algorithm include low-frequency (LFC; 0.01–0.1 Hz), high-frequency (HFC; 0.1–0.4 Hz), and very low-frequency (vLFC; 0.001–0.01 Hz) couplings, and an elevated LFC-broad band (eLFC_BB_) that is a sleep fragmentation signal biomarker (Thomas et al., 2005). HFC/LFC covary more strongly with an electroencephalographic (EEG) non-cyclic alternating pattern (n-CAP) and CAP, respectively (Thomas et al., 2005), than conventional N3/N2—although most of N3 is HFC, and much of HFC occurs during N2.

Stable-NREM sleep (HFC) is associated with several desirable sleep characteristics, including increased absolute and relative delta power (Thomas et al., 2014), a consolidated NREM sleep <1-Hz slow oscillation, temporally stable breathing, stable arousal thresholds, normal arterial oxygen (O_2_) and carbon dioxide (CO_2_) concentrations, and BP dipping (Wood et al., 2020). Unstable NREM (LFC) is characterized by features opposite of stable-NREM (HFC), and ineffective (fragmented) REM sleep takes on LFC coupling signatures, while wake or effective REM sleep shows vLFC pattern (Thomas et al., 2005). HFC covaries better with relative than absolute EEG slow-wave power and is thus less constrained by the “loss” of slow-wave sleep (SWS) with age (Thomas et al., 2014). Specific spectrographic signatures of fragmented sleep (elevated LFC narrow band, eLFCNB) are biomarkers of strong chemoreflex effects on sleep respiration (Thomas et al., 2007), identifying areas of sleep with central apneas and periodic breathing. BP dipping occurs only during periods of HFC (Wood et al., 2020), consistent with the demonstration that non-CAP is the EEG correlate of BP dipping (Iellamo et al., 2004). LFC is associated with hypertension and stroke (Thomas et al., 2009), while HFC is reduced in depression (Yang et al., 2011), heart failure (Yeh et al., 2008), and fibromyalgia syndrome (Thomas et al., 2010). HFC is an independent determinant of the glucose disposition index (Pogach et al., 2012). Pre- and posttreatment effects in sleep apnea are captured via changes in HFC/LFC (Lee et al., 2014). An integrated metric, the Sleep Quality Index (SQI), which is heavily weighted by stable-NREM sleep, is associated with desirable directions of metabolic health and blood pressure (Magnusdottir et al., 2020, 2021, 2022).

#### 2.4.3 HR and CPC analysis

The PPG signal from each polygraphy recording in the HeartBEAT data was processed through the SleepImage System algorithms. A software module (“HR module”) was developed for evaluating the HR data collected during the sleep study, which was then intersected with the sleep-state output from the SleepImage System: (a) A 2-sample-per-second resampled NN series was evaluated by cropping the entire series from sleep onset to sleep conclusion. Then, a linear trendline was fitted to generate a slope coefficient, the associated *p*-value, and an *R*^2^ for model fit. While this method may provide insight that some clinical professionals may value, it was not expected to have much explanatory power, as it attempts to describe HR as a linear trend over the course of the sleep period, lumping all sleep states together. The disproportionate effect of sleep stage on BP dynamics has been explained in the literature (Stein and Pu, 2012). (b) HR rate statistics for each CPC sleep state classification were then calculated using the same 2-sample-per-second resampled NN series.

#### 2.4.4 Primary endpoint

For this analysis, the following variables of interest were extracted from the HeartBEAT data set: (a) MAP non-dipping and dipping, (b) systolic blood pressure (SBP) non-dipping and dipping, and (c) diastolic blood pressure (DBP) non-dipping and dipping, where non-dippers were defined as patients who did not demonstrate a decrease in BP ≥10% from wake to sleep. The primary endpoint was to evaluate (a) the relationship between HR during stable and unstable CPC sleep states and BP and (b) if sleep evaluation combined with this HR module can be utilized to stratify patients to identify patients more likely to have non-BP dipping during sleep for further evaluation.

### 2.5 Statistical analysis

The primary endpoint, questioning if the HR module can be utilized to identify BP dipping during sleep, was evaluated using a logistic regression, regressing HR parameters from the HR module on the dipping and non-dipping variables extracted from the HeartBEAT data, controlling for gender, age, and race to assess the predictive power on BP dipping.

Basic summary statistics, such as counts, are presented, along with means and standard errors (in parentheses) for gender, race, age, and body mass index (BMI). A one-way analysis of covariance was utilized to compare non-dippers and dippers controlling for age, gender, BMI, and site identifier. Means and standard errors for the HR parameters are presented, along with the *p*-values for pairwise comparison between the groups. The analysis was performed using Stata version 15.1.

## 3 Results

### 3.1 Study population

Included in this analysis of the pleth signal from the HeartBEAT data (Gottlieb et al., 2014) are both baseline and follow-up recordings with signal quality defined as average successful peak detection on the overnight recording of no <70% for analysis to evaluate HR during sleep and on data describing dipping and non-dipping BP status. The data set contained sleep recordings with acceptable pleth-signal quality from 302 patients at baseline and 267 at follow-up, or a total of 569 sleep recordings (Supplementary Table S1).

### 3.2 Primary outcome measures

First, the relationship between the CPC-sleep-state analysis and the software HR module and dipping or non-dipping status of BP was evaluated (Table 1). The most significant difference in minimum HR (HR_min_) was observed when comparing HR for dippers (56 ± 4 BPM) and non-dippers (59 ± 4 BPM) during DBP and stable-NREM sleep (*p* < 0.0001).

**Table 1:** Heart rate (HR) software module (“HR parameters”) for dippers and non-dippers, beats per minutes (BPM).

	**Average HR (BPM) measured during diastolic blood pressure (mm/Hg)**			**Average HR (BPM) measured during systolic blood pressure (mm/Hg)**			**Average HR (BPM) measured during mean arterial blood pressure (mm/Hg)**		
	**Non-Dip** ^a^	**Dip** ^a^	***p*** **-value** ^b^	**Non-Dip** ^a^	**Dip** ^a^	***p*** **-value** ^b^	**Non-Dip** ^a^	**Dip** ^a^	***p*** **-value** ^b^
**Sleep**									
Mean HR	66.4 (±5.5)	64.7 (±5.5)	0.063	66.0 (±5.5)	65.1 (±5.5)	0.301	66.6 (±5.5)	65.1 (±5.5)	0.090
Min HR	55.6 (±2.6)	54.31 (±2.6)	0.003	55.6 (±2.6)	54.6 (±2.6)	0.016	55.8 (±2.6)	54.6 (±2.6)	0.003
**Wake**									
Mean HR	69.2 (±5.7)	67.8 (±5.6)	0.126	69.1 (±5.7)	68.1 (±5.6)	0.293	69.6 (±5.7)	68.1 (±5.6)	0.100
Min HR	57.5 (±3.0)	55.9 (±3.0)	0.001	57.7 (±3.0)	56.2 (±3.0)	0.003	57.871 (±3.0)	56.3 (±3.0)	0.001
**Stable NREM**									
Mean HR	67.2 (±7.4)	64.3 (7.3)	0.017	66.1 (±7.4)	64.9 (±7.34)	0.318	66.6 (±7.4)	64.6 (±7.3)	0.150
Min HR	58.6 (±4.1)	56.3 (4.1)	<0.0001	57.6 (±4.2)	56.74 (±4.1)	0.189	58.25 (±4.2)	56.7 (±4.1)	0.022

HR, heart rate; NREM, non-rapid eye movement sleep; BPM, heart beats per minute.

^a^Values are mean ± standard deviation.

^b^Difference between groups (p-value).

Table 2 presents the result from different logit models regressing variables of interest (HR metrics; HR_min_ during sleep and HR_min_ during stable-NREM sleep), controlling for age and race on the indicator variables for diastolic, systolic, and MAP non-dipping. BMI was explored as a control but did not add to the explanatory/predictive power of any of the models. The results are presented as coefficient estimates from the logit regression with *p*-values in parentheses. The strongest associations were observed when including HR_min_ during stable-NREM sleep. Higher HR_min_ were associated with a higher likelihood of being a non-dipper, with an increase of 1 beat per minute in HR_min_ during stable NREM being associated with an approximate 4.4% increase in the probability of being a non-dipper (*p* = 0.001). Being African American and increased age were found to be factors that increased the likelihood of being a non-dipper, while gender did not seem to be a significant predictor. For the sake of brevity, the mean HR in stable-NREM sleep was not included in the results, although it showed a statistically significant effect on diastolic non-dipping, in favor of the minimum, which had a higher coefficient. The variance inflation factor (VIF) was calculated to investigate the presence of collinearity. The average VIF did not exceed 1.08 for any of the models, with the highest calculated VIF of 1.14 for an individual variable. This indicates that multicollinearity is not of concern with the chosen independent variables.

**Table 2:** Logistic regression models regressing the HR parameter on diastolic, systolic, and mean arterial blood pressure (MAP) non-dip indicators.

	**Diastolic non-dipping blood pressure** ^**a**^		**Systolic non-dipping blood pressure^a^**	**MAP non-dipping blood pressure** ^**a**^	
Sleep_min_		0.049 (0.010)	0.041 (0.035)		0.049 (0.010)
Stable NREM_min_	0.044 (0.001)			0.026 (0.032)	
Male	0.092 (0.674)	0.117 (0.591)	0.030 (0.891)	0.153 (0.483)	0.193 (0.378)
**Race**					
African American	0.744 (0.009)	0.720 (0.012)	1.013 (0.001)	0.960 (0.001)	0.921 (0.002)
Other	0.086 (0.815)	−0.002 (0.995)	0.007 (0.983)	0.195 (0.592)	0.117 (0.749)
Age	0.019 (0.146)	0.023 (0.073)	0.035 (0.008)	0.026 (0.049)	0.032 (0.016)
Const.	−4.205 (0.000)	−4.572 (0.001)	−4.400 (0.002)	−3.532 (0.002)	−5.010 (0.000)

NREM, non-rapid eye movement sleep; MAP, mean arterial pressure.

^a^Values are the logistic regression coefficients with p-values in parenthesis.

Second, the cohort was stratified based on the “HR parameter” into HR dippers and HR non-dippers (Table 3). At baseline, fewer HR non-dippers used calcium-channel blockers (10.7%, *p* = 0.047) and diuretics (10.9%, *p* = 0.044) than HR-dippers. Additionally, HR non-dippers presented with a higher mean MAP when awake of 2.2 mmHg (*p* = 0.046) than HR-dippers. Comparing HR dippers and non-dippers and focusing on BP, HR non-dippers have significantly higher average DBP (DBP^ave^; 85.3 vs. 83.8 mmHg, *p* = 0.035); DBP^sleep^ (74.6 vs. 72.7 mmHg, *p* = 0.018); and DBP^wake^ (90.8 vs. 89.0 mmHg, *p* = 0.024). Additionally, they have a higher average MAP (MAP^ave^; 89.7 vs. 88.1 mmHg, *p* = 0.050); MAP^sleep^ (79.4 vs. 77.4 mmHg, *p* = 0.036); and MAP^wake^ (95.2 vs. 93.3 mmHg, *p* = 0.031), respectively (Table 4).

**Table 3:** Baseline characteristics: heart rate dippers vs. non-dippers.

	**Heart rate** ^**a**^			
	**All**^a^ **(*****n*** = **302)**	**HR dippers**^a^ **(*****n*** = **143, 47.4%)**	**HR non-dippers**^a^ **(*****n*** = **159, 52.6%)**	**HR dippers compared to HR non-dippers (** ***p*** **-values)** ^b^
**Characteristics**			
Age (years)	63.0 (±0.410)	63.1 (±0.617)	63.0 (±0.585)	0.910
Body mass index (BMI; kg/m^2^)	34.4 (±0.358)	34.9 (±0.539)	33.7 (±0.511)	0.103
Neck circumference (cm)	42.2 (±0.211)	42.3 (±0.313)	42.0 (±0.297)	0.561
Waist/hip ratio	1.0 (±0.004)	1.0 (±0.006)	1.0 (±0.006)	0.091
African American (%)	13.2	13.3	12.6	0.855
Caucasian (%)	79.2	78.3	79.9	0.741
Ever smoked (%)	62.9	6.3	11.3	0.127
**Coexisting conditions (%)**			
Prior myocardial infarction	22.0	22.3	23.3	0.532
Diabetes mellitus	42.5	44.3	40.9	0.554
Dyslipidemia	77.8	77.8	78.6	0.874
Hypertension	82.8	85.7	80.5	0.233
**Obstructive sleep apnea (%)**			
Moderate (AHI 15–30)	98.4	97.2	99.3	0.141
Severe (AHI ≥ 30)	29.6	29.4	29.6	0.971
**Medication use (%)**			
ACE or ARB	69.8	73.4	64.8	0.106
Any beta-blockers	67.3	65.7	66.7	0.865
Calcium-channel blocker	30.1	37.1	26.4	0.047
Medication for diabetes	39.3	42.7	35.8	0.227
Diuretic	37.7	42.7	31.8	0.044
Lipid-lowering medication	89.0	86.0	91.8	0.107
**Questionnaires**			
Epworth Sleepiness Scale	8.9 (±0.204)	9.1 (±0.304)	8.8 (±0.288)	0.431
Patient Health Questionnaire-9 (PHQ-9)	5.5 (±0.279)	5.5 (±0.420)	5.6 (±0.399)	0.971
PHQ-9 depression severity	0.7 (±0.054)	0.8 (±0.080)	0.7 (±0.076)	0.687
**Sleep measures**			
Sleep Quality Index (SQI)	41 (±0.910)	41 (±1.324)	40 (±1.255)	0.489
Apnea-Hypopnea Index (AHI)	33 (±0.555)	33 (±0.809)	33 (±0.764)	0.935
Stable sleep (%)	27 (±1.030)	28 (±1.498)	26 (±1.420)	0.352
Unstable sleep (%)	50 (±0.982)	49 (±1.428)	51 (±1.354)	0.476
Fragmentation (eLFC_BB:_%)	26 (±0.945)	26 (±1.375)	26 (±1.304)	0.925
Periodicity (e-LFC_NB:_%)	3 (±0.276)	3 (±0.401)	3 (±0.381)	0.910
**Blood pressure measures (mm/Hg)**			
Average diastolic pressure, all readings	71 (±0.456)	70 (±0.684)	71 (±0.646)	0.101
Average diastolic pressure, sleep	65 (±0.490)	64 (± 0.231)	65 (±0.685)	0.184
Average diastolic pressure, wake	73 (±0.742)	72 (±0.742)	74 (±0.701)	0.067
Average systolic pressure, all readings	124 (±0.833)	123 (±1.262)	125 (±1.191)	0.160
Average systolic pressure, sleep	116 (±0.943)	115 (±1.415)	117 (±1.330)	0.223
Average systolic pressure, wake	128 (±0.844)	126 (±1.280)	129 (±1.208)	0.102
Average mean arterial pressure, sleep	83 (±0.573)	82 (±0.855)	84 (±0.804)	0.138
Average mean arterial pressure, wake	92 (±0.523)	91 (±0.789)	93 (±0.745)	0.046

HR, heart rate; ACE, angiotensin converting enzyme; ARB, angiotensin receptor blocker.

^a^Values are mean ± standard deviation for continuous variables and percentages for categorical variables.

^b^Difference between groups (p-value).

**Table 4:** Heart rate dipper vs.. non-dipper: blood pressure and biochemical measures.

	**HR-dipper^a^ (*n* = 143, 47.4%)**	**HR-non-dippera (*n* = 159, 52.6%)**	***p*-values^b^**
**Medication use (%)**			
ACE or ARB	76	65	0.008
Any beta-blockers	59	56	0.455
Calcium-channel blocker	12	2	0.017
Medication for diabetes	28	19	0.028
Diuretic	26	17	0.031
Lipid-lowering medication	35	35	0.953
**Blood pressure measures (mm/Hg)**			
Average diastolic blood pressure	84 (±3.183)	85 (±3.164)	0.035
Average diastolic pressure, sleep	73 (±3.459)	75 (±3.436)	0.018
Average diastolic pressure, wake	89 (±3.392)	91 (±3.371)	0.024
Average systolic pressure	106 (±5.723)	108 (±5.688)	0.230
Average systolic pressure, sleep	94 (±6.415)	96 (±6.371)	0.145
Average systolic pressure, wake	112 (±5.854)	114 (±5.818)	0.175
Average mean arterial pressure	88 (±3.568)	90 (±3.546)	0.050
Average mean arterial pressure, sleep	77 (±4.040)	79 (±4.013)	0.036
Average mean arterial pressure, wake	93 (±3.713)	95 (±3.690)	0.031

HR, heart rate; ACE, angiotensin converting enzyme; ARB, angiotensin receptor blocker.

^a^Values are mean ± standard deviation for continuous variables and percentages for categorical variables.

^b^Difference between groups (p-value).

Finally, the subgroup that received the CPAP therapy was stratified based on dipping status (HR dipper or HR non-dipper) at baseline and at a 12-week follow-up (Table 5). When comparing participants who were HR non-dippers at baseline and HR dippers at follow-up, significant improvements were observed in depression severity measured using the Patient Health Questionnaire-9, −3.1 (*p* = 0.005), and the health and quality-of-life indicators that were evaluated using the Short Form (36) Health Survey: (a) vitality, 12.8 (*p* = 0.036); (b) physical functioning, 20.1 (*p* = 0.003); and (c) emotional functioning, 16.5 (*p* = 0.005); social functioning, 17.4 (*p* = 0.007); and mental health and emotional wellbeing, 13.9 (*p* = 0.001).

**Table 5:** Heart rate dipper vs. non-dippers: comparison of characteristics.

	**Baseline**		**Follow-Up**		***p*** **-values** ^**b**^			
	**I. HR-dipper**^a^ **(*****n*** = **45, 45.9%)**	**II. HR-non-dipper**^a^ **(*****n*** = **53, 54.1%)**	**III. HR-dipper**^a^ **(*****n*** = **42, 51.2%)**	**IV. HR-non-dipper**^a^ **(*****n*** = **40, 48.8%)**	**III vs. I**	**IV. vs. I**	**III vs. II**	**IV vs. II**
**Characteristics**								
Age (years)	63.3 (±1.025)	62.5 (±0.618)	64.0 (±1.074)	62.5 (±1.087)	0.966	0.951	0.729	1.000
Body mass index (BMI; kg/m^2^)	33.6 (±0.769)	33.5 (±0.708)	32.5 (±0.815)	33.9 (±0.815)	0.757	0.997	0.795	0.898
Neck circumference (cm)	42.2 (±0.518)	41.5 (±0.478)	41.1 (±0.550)	42.0 (±0.550)	0.477	0.944	0.948	0.906
Waist/hip ratio	1.0 (±0.009)	1.0 (±0.008)	1.0 (±0.009)	1.0 (±0.009)	0.998	1.000	0.848	0.742
African American (%)	8.9	3.8	9.5	2.5	0.999	0.613	0.654	0.994
Caucasian (%)	82.2	86.8	78.6	85.0	0.969	0.986	−0.714	0.996
**Coexisting conditions (%)**								
Prior myocardial infarction	15.6	18.9	14.3	25.0	0.999	0.680	0.941	0.876
Diabetes mellitus	37.2	43.4	57.1	37.5	0.252	0.975	0.537	0.941
Dyslipidemia	79.1	83.0	78.6	67.5	1.000	0.590	0.955	0.291
Hypertension	72.1	84.9	81.0	67.5	0.765	0.959	0.968	0.200
**Medication use (%)**								
ACE or ARB	68.9	66.0	78.0	60.0	0.800	0.817	0.604	0.926
Any beta-blockers	57.8	62.3	58.5	57.5	1.000	1.000	0.984	0.968
Calcium-channel blocker	40.0	26.4	26.8	27.5	0.549	0.710	1.000	0.999
Medication for diabetes	35.6	33.9	48.8	25.0	0.575	0.740	0.445	0.807
Diuretic	28.9	30.2	41.5	35.0	0.611	0.934	0.664	0.963
Lipid-lowering medication	93.3	88.7	92.7	85.0	1.000	0.584	0.920	0.938
**Questionnaires**								
Epworth sleepiness scale score	8.2 (±0.554)	8.1 (±0.511)	8.6 (±0.574)	8.2 (±0.588)	0.972	1.000	0.927	1.000
Patient Health Questionnaire-9 (PHQ-9)	4.8 (±0.658)	5.7 (±0.606)	2.6 (±0.681)	4.6 (±0.698)	0.119	1.000	0.005	0.657
PHQ-9 Depression severity	0.6 (±0.123)	0.8 (±0.113)	0.2 (±0.127)	0.6 (±0.130)	0.233	0.999	0.01	0.746
**Short Form (36) Health Survey**								
SF-vitality	54.8 (±3.370)	54.1 (±3.105)	66.9 (±3.530)	57.0 (±3.574)	0.067	0.969	0.036	0.928
SF-physical functioning	74.4 (±4.190)	65.5 (±3.861)	86.1 (±4.390)	65.6 (±4.445)	0.221	0.474	0.003	1.000
SF-general health perceptions	62.6 (±3.345)	56.4 (±3.082)	66.5 (±3.504)	60.9 (±3.548)	0.853	0.984	0.138	0.778
SF-emotional functioning	81.3 (±3.514)	75.6 (±3.238)	92.1 (±3.681)	76.7 (±3.727)	0.152	0.803	0.005	0.997
SF-social functioning	81.1 (±3.797)	73.8 (±3.499)	91.2 (±3.978)	76.6 (±4.027)	0.264	0.844	0.007	0.997
SF-mental health/emotional wellbeing	74.0 (±2.567)	72.2 (±2.365)	86.1 (±2.689)	72.6 (±2.722)	0.007	0.983	0.001	1.000
**Sleep measures**								
Sleep Quality Index (SQI; 0–100)	41 (±2.538)	40 (±2.339)	42 (±2.627)	41 (±2.692)	0.995	1.000	0.957	0.994
Apnea-Hypopnea Index (AHI)	32 (±1.774)	32 (±1.635)	20 (±1.836)	25 (±1.882)	<0.001	0.042	<0.001	0.013
**Blood pressure measures (mm/Hg)**								
Average diastolic blood pressure, all readings	73 (±1.117)	71 (±1.028)	69 (±1.157)	70 (±1.187)	0.063	0.382	0.499	0.963
Average diastolic pressure, sleep	68 (±1.236)	66 (±1.135)	63 (±1.282)	65 (±1.315)	0.062	0.534	0.488	0.996
Average diastolic pressure, wake	76 (±1.185)	74 (±1.090)	71 (±1.228)	73 (±1.259)	0.074	0.552	0.461	0.989
Average systolic pressure, all readings	126 (±2.014)	122 (±1.853)	123 (±2.087)	122 (±2.140)	0.816	0.685	0.990	1.000
Average systolic pressure, sleep	118 (±2.378)	115 (±2.184)	115 (±2.466)	115 (±2.530)	0.791	0.858	1.000	1.000
Average systolic pressure, wake	130 (±2.026)	126 (±1.864)	127 (±2.099)	126 (±2.152)	0.745	0.704	0.990	0.995
Average mean arterial pressure, sleep	85 (±1.488)	83 (±1.366)	82 (±1.543)	83 (±1.583)	0.288	0.724	0.860	1.000
Average mean arterial pressure, wake	94 (±1.264)	92 (±1.163)	90 (±1.310)	92 (±1.343)	0.238	0.700	0.859	1.000

HR, heart rate; ACE, angiotensin converting enzyme; ARB, angiotensin receptor blocker.

^a^Values are mean ± standard deviation for continuous variables and percentages for categorical variables.

^b^P-values based on difference between groups at baseline and follow-up.

## 4 Discussion

The analysis of HR across the night in relation to CPC sleep state, HR, and BP showed the following statistically significant results: (a) lower HR_min_ during sleep in participants who demonstrated diastolic, systolic, and MAP dipping when compared to non-dippers; (b) lower HR_min_ during wake *within* the sleep period in participants who demonstrated diastolic-, systolic-, and MAP dipping compared to non-dippers; (c) lower HR_min_ and HR_mean_ during stable-NREM sleep (HFC) in those who demonstrated diastolic dipping compared to non-dippers; (d) the strongest associations were observed when including HR_min_ during stable-NREM sleep (HFC); (e) when utilizing the HR module to stratify the cohort based on HR dipping and non-dipping, participants with non-dipping of HR had significantly higher MAP and DBP when comparing all readings, readings during sleep, and readings during wake; and (f) in the subgroup of participants who received CPAP-therapy, participants with a HR non-dipping pattern at baseline and HR dipping pattern at follow-up (i.e., treatment responders based on heart rate profiles), significantly improved their subjective mental and physical functioning. The results overall suggest that HR dipping in stable-NREM sleep/HFC is a desirable biological characteristic.

HR kinetics during sleep seems to provide indirect information about BP during sleep, an important cardiovascular health variable. HR is readily available through most systems that track sleep oximetry and could allow for risk stratification; individuals with a non-dipping HR pattern could be directed to selectively undergo conventional ABPM. The finding that HR was specifically influenced by stable-NREM sleep (HFC) as estimated through the pleth spectrogram was not surprising. Autonomic physiology presents an important window into sleep; for example, hemodynamics, HRV, and respiration are markedly dependent on the sleep stage, with vagal dominance, stable breathing, and BP reductions (“dipping”) during SWS/N3 (Javaheri and Redline, 2012). Furthermore, standard reporting of EEG-based stages as a percentage of sleep time is an insensitive metric of sleep fragmentation (Bianchi and Thomas, 2013). Most HSATs do not provide EEG stages, although machine learning applied to ECG and respiratory signals can approximate deep sleep. The ability to evaluate HR dynamics in relation to sleep state without the need for extensive PSG has practical advantages.

A reduction in BP during sleep (BP dipping) is considered a BP-related biomarker of healthy sleep (Routledge et al., 2007; Salles et al., 2016). There is a progressive reduction of BP from wake through SWS, with an increase in REM sleep or transiently in association with arousals. The HR profile follows this basic scheme and is the highest in REM sleep and unstable-NREM sleep or during arousals and lowest in conventional N3 (Javaheri and Redline, 2012). In a prior study by our group, we used PSG with beat-to-beat BP monitoring, ECG-derived CPC analysis, and quantified delta power and the rate of occurrence of the <1-Hz slow oscillation. We found that BP dipping occurred only during periods of stable NREM (HFC), concomitant with slow oscillation/delta power-enriched NREM sleep. HR was lowest in N3, but the small sample size of 11 subjects perhaps explained the lack of HR dipping during HFC; however, the current analysis shows the predicted dip in HR. Mechanisms associated with rising slow wave/delta power and a high grade of electrocortical synchrony are likely the drivers of an integrated response of BP, HR, and stable breathing. Even in conditions associated with fragmented sleep, such as sleep apnea, delta power, and vagal HRV dominance tends to ebb and flow in a correlated manner (Jurysta et al., 2006; Wood et al., 2020).

There is substantial variability in sleep quality in individuals with similar severities of sleep apnea. Such differences may be quantified by subjective symptoms e.g., questionnaire such as the Insomnia Severity Index (Bastien et al., 2001); conventional criteria e.g., N1, N3, total sleep time, sleep efficiency; EEG-based methods such as the odds ratio product, which estimates sleep depth continuously (Younes et al., 2015; Younes, 2023); and ECG/PPG CPC spectrograms and the SQI (Thomas et al., 2014; Hilmisson et al., 2019; Magnusdottir et al., 2020). BP during sleep is another useful measure, while HR analysis could provide a complementary metric for sleep quality.

There are sleep and circadian influences on BP and HR control. Even in forced desynchrony experiments, both metrics are low in the biological night when body temperature is low and melatonin is high. Thus, there are both sleep and circadian components to BP and HR dipping, and a loss of this pattern can occur from either sleep or circadian factors. Any case of sleep fragmentation can flatten or even reverse BP and HR during the biological night. OSA can affect sleep quality and cause non-dipping BP by autonomic dysfunction, transient surges associated with arousals, the upregulation of neurohumoral systems, oxidative stress, and a general lowering of sleep depth. However, when there is non-dipping of either BP or HR during stable (unfragmented) sleep of which HFC is a good biomarker, it likely reflects abnormal autonomic regulation as many other drivers are less active during this state (e.g., breathing and oxygenation are stable). Profiles of HR may be useful when following treatment of sleep apnea, especially with therapies with residual apnea. While CPAP when used can largely normalize breathing, other increasingly used therapies, such as weight loss, hypoglossal nerve stimulation, and oral appliances, are more likely to have residual apnea. Partial CPAP use will also demonstrate residual apnea. HR profiles could be one way to assess the impact of residual disease as successful therapy could be expected to improve HR dipping and even convert a non-/reverse dipper to a dipper as demonstrated in this analysis when looking at the subgroup treated with CPAP.

This analysis has some limitations, including (a) the study population was selected for presence of CV disease or risk and does not readily generalize to the range of medical backgrounds on which sleep apnea occurs; (b) classic sleep staging was not available or possible; (c) only one night of recording was available at any given time point; (d) HR can be modified by numerous factors including stress, anxiety, and pain, the impact of which on the type of analysis we performed is unknown; (e) the implications of HR-pattern analysis for disease prognostics, well established for conventional ambulatory blood pressure, are unknown; (f) the impact of drugs such as beta-blockers or antihypertensives in general on the noted patterns need to be established, and it is plausible that both attenuation or amplification of the HR response during sleep may occur based on cardiovascular functional status; and (g) conditions such as heart failure, advanced renal disease, post-cardiac transplant, or advanced autonomic neuropathy are likely to have relatively unchanging HR across the night and may not allow this analysis. Heart rate analysis would be invalidated by atrial fibrillation and during fixed-rate cardiac pacing for bradyarrhythmias, while other modes of pacing may still allow analysis, but that needs to be directly demonstrated. The parent study design and our current analysis cannot determine if HR is an independent risk factor (beyond nocturnal BP) for cardiovascular outcomes. Additionally, a generalization of its potential usefulness to non-apnea conditions such as insomnia or restless legs and their treatments cannot be determined.

In conclusion, HR analytics in relation to the sleep period and the CPC spectrogram-estimated sleep states can provide novel and potentially clinically useful information on autonomic health. HR dipping (or not) may be a useful screener of BP dipping/non-dipping and identify individuals who may benefit from formal assessment of ambulatory BP and/or evaluate the efficacy of various therapies. Such a stepped approach may enable a more practical, cost-effective, and applicable approach to diagnosing MH.

## Data availability statement

Publicly available datasets were analyzed in this study. This analysis was made possible by the National Sleep Research Resource, with access to the HeartBEAT-database—https://www.sleepdata.org/datasets/heartbeat.

## Ethics statement

The studies involving humans were approved by Brigham & Women's Hospital and Case Western Reserve University. The studies were conducted in accordance with the local legislation and institutional requirements. The participants provided their written informed consent to participate in this study.

## Author contributions

HH conceptualized and designed the analysis, analyzed the data and conducted the statistical analysis, interpreted the data output, and reviewed, edited, and approved the overall content of the final manuscript. RT and SM wrote the manuscript, interpreted the data, and reviewed, edited, and approved the overall content of the final manuscript. All authors approved the final version of the manuscript and agreed to be accountable for all aspects of the study in ensuring that questions related to the accuracy or integrity of any part of the study are appropriately investigated and resolved.
