# Supplementary material for: Cardiopulmonary coupling-calculated sleep stability and nocturnal heart rate kinetics as a potential indicator for cardiovascular health: a relationship with blood pressure dipping
Source: Front Sleep. 2024 Aug 1;3:1230958. doi: 10.3389/frsle.2024.1230958 (PMC12713940; doi:10.3389/frsle.2024.1230958)
Supplement: Supplementary file 1 [file Table_1.docx]

| **Supplement 1. Baseline demographics of patients included in the analysis.** | | |
| --- | --- | --- |
|  |  |  |
|  | Baseline (302) | Follow-Up (267) |
| Male | 220 | 198 |
| Race |  |  |
| Caucasian | 239 | 213 |
| African American | 40 | 36 |
| Other | 19 | 18 |
| Age | 63.05 (7.20) | 63.23 (7.30) |
| BMI | 34.00 (5.67) | 34.13 (6.04) |
| BMI: body mass index |  |  |
